# Promoter methylation changes in the placenta involved in the relationship between prenatal depression and small for gestational age

**DOI:** 10.1186/s12884-022-05066-3

**Published:** 2022-10-02

**Authors:** Jianhui Yang, Aitong Xu, YuMin Zhang, Jiahui Deng, Xuemei Lin, Lili Xie, Xiaochun Deng, Honglin Liu, Peishan Chen, Yuejun Huang

**Affiliations:** 1 Department of Neonatology, Second Affiliated Hospital of Shantou University Medical College, North Dongxia Road, Shantou, 515041 Guangdong China; 2 Huizhou Central People’s Hospital, North Erling Road, Huizhou, 516003 Guangdong China; 3 Shantou University Medical College, Xinling Road, Shantou, 515041 Guangdong China; 4 Department of Obstetrics, Second Affiliated Hospital of Shantou University Medical College, North Dongxia Road, Shantou, 515041 Guangdong China

**Keywords:** Maternal depression, Small for gestation age, CRH, HSD11β2, DIO3, Methylation, Prenatal depression, Hormone, Placenta

## Abstract

**Background:**

Recent studies suggest that the incidence of small for gestational age (SGA) birth related to maternal depression, but the mechanism is unclear. The aim of this study was to explore the changes of promoter methylation in the placenta which may be involved in the relationship between prenatal depression and SGA.

**Methods:**

Three hundred forty-five pregnant women were enrolled in this prospective cohort study. Perinatal emotion and sleep quality in the second and third trimesters were assessed using self-rating depression scale, self-rating anxiety scale, and Pittsburgh sleep quality index. According to the exposure (depressed emotion of mother) and outcome (SGA), the placentas were divided into four groups. Methylation of the promoter regions of the placental CRH, HSD11β2, SLA16A10, DIO3, and MTNR1B genes was determined using next generation sequencing based on bisulfite sequencing PCR.

**Results:**

There were 97 (28.1%) and 95 (27.5%) pregnant women who had depression in the second trimester and third trimester, respectively. Thirty-five pregnant women had an SGA birth. The incidence of SGA births in this prospective cohort was 10.1%. The risk factors of SGA birth were low BMI of pregnancy women (RR = 0.71, 95%CI = 0.54 ~ 0.92), hypertensive disorder complicating pregnancy (HDCP, RR = 4.7, 95%CI = 1.18 ~ 18.72), and maternal depression in the second trimester (RR = 3.71, 95%CI = 1.31 ~ 12.16). We found that the CRH and HSD11β2 methylation levels were higher in the depression group than those in the non-depression group. Methylation levels of DIO3 were higher in SGA group than that in the non-SGA group. Higher methylation levels of CRH correlated with higher methylation levels of DIO3 in the placenta.

**Conclusions:**

Maternal depression in the second trimester may lead to the changes of methylation levels in the promoter region of CRH and HSD11β2 gene, while the changes of methylation of DIO3 in subsequent could related to SGA. This study suggests that maternal depressed emotion during pregnancy may result in SGA due to the epigenetic changes of placenta.

## Background

Depression and anxiety are the most common emotional problem during pregnancy, influencing 10% to 40% pregnant woman [1, 2]. Maternal depressed emotion is one of the risk factors of low birth weight [3], which could result in higher incidence of small for gestational age (SGA). But how does a mother's emotion during pregnancy affect the physical development of the fetus is unknown. Depression could increase the level of glucocorticoids due to the abnormal function of hypothalamic–pituitary–adrenal (HPA) axis [4]. Placenta is an important regulator of fetal growth and development, which involved in the synthesis of growth factors and hormones during pregnancy [5]. The placenta regulates fetal exposure to glucocorticoids by converting active glucocorticoids into their inactive metabolites via 11β-hydroxysteroid dehydrogenase type 2 (HSD11β2) [6]. Excessive maternal glucocorticoids can, however, exceed the barriers functional capacity and allow an influx of glucocorticoids into the fetal circulation, which can impair fetal growth and program offspring disease [7].

Thyroid hormone secreted by hypothalamus-pituitary-thyroid (HPT) axis, be indicated closely related to birth weight [8], which can mutually regulate to the HPA axis hormones by mediating corticotropin-releasing hormone (CRH) [9]. Solute carrier family 16 member 10 (SLC16A10) gene encode monocarboxylate transporters that highly active and specific for thyroid hormones [10]. The deiodinase 3 (DIO3) gene encode D3, which is largely expressed in the placenta and is linked to prenatal growth in humans [11]. In addition, depression can affect sleep quality. Sleep is regulated by melatonin, a key hormone determining the sleep–wake cycle in humans [12]. Melatonin also can regulate the HPA axis [13]. In animal studies, melatonin treatment has been shown to reduce abnormal elevated corticosterone levels to normal [14], and adverse stress can cause melatonin secretion disorders. Melatonin receptor 1B (MTNR1B) gene encode high-affinity receptors of melatonin, which expresses in the placenta during pregnancy.

Placental epigenetic, which are susceptible to maternal environment, including physical and psychological disorders [15], can functionally regulate gene expression and phenotype [16]. Hence it has been suggested that placental epigenetic could serve as integrated measures of the experienced environment and play an important role in maintaining maternal health and promoting fetal growth and development in utero [17]. According to the above mentioned, we hypothesized that epigenetic modification in the placenta is the potential mechanism mediating the relationship between SGA birth and maternal emotion during pregnancy. Therefore, we will use a prospective cohort study of pregnancy women to analyze the relationship between SGA and maternal emotion during pregnancy, and then we will detect methylation changes in the promoter region of the target genes in the placenta, including CRH, HSD11β2, SLC16A10, DIO3, and MTNR1B.

## Methods

### Ethics approval and consent to participate

This is a prospective cohort study conducted on pregnant women. The study protocol was approved by the research institute’s committee of human research in the Second Affiliated Hospital of Shantou University Medical College (NO.2016027) and abided by the standards of the Declaration of Helsinki. All methods were carried out in accordance with relevant guidelines and regulations. Informed consent was obtained from pregnancy women.

### Participants

We collected data of pregnant women in the obstetrics clinic from January 2018 to January 2020 for prospective analysis. The inclusion criteria of pregnant women were as follows: (1) singleton pregnancy, (2) intended to have regular antenatal care and give birth in our hospital, and (3) be able to understand the relevant scale options in this subject. The pregnant women were excluded from this study if they and the baby’s father met the following criteria: (1) had thyroid, liver, kidney, lung or heart disease before and after pregnancy, (2) had depression, anxiety disorder, somnipathy, schizophrenia, mania, dissociative personality disorder, and other mental disorders prior to pregnancy, and (3) TORCH infection.

### Questionnaire survey

Participants were required to complete a questionnaire, at the first visit for antenatal care, which was used to collect variables including the age of the pregnant women, pre-pregnancy body mass index (BMI), education level, economic level, history of adverse pregnancy, pregnancy complications, and life style (smoking, drink, diet).

### Data collection

Data collection was performed after delivery, including age of mother, BMI, exposure to passive smoking, adverse pregnancy history, education level, economic level, vegetarianism, pregnancy anemia, hypertensive disorder complicating pregnancy (HDCP), pregnancy diabetes, pregnancy hypothyroidism, method of delivery, preterm birth, gestational age, birth weight, and gender. Adverse pregnancy history included spontaneous abortion, induced abortion, stillbirth, and preterm birth [18]. Education level was divided into with and without a college education. Economic level was divided into two levels according to the minimum taxable income, which is 5000 yuan in China. Diagnosis of pregnancy anemia [19], hypertensive disorder complicating pregnancy (HDCP) [20], pregnancy diabetes [21], uterine infection [22], and placenta previa [23] were according to the diagnosis guidance.

### Assessment of maternal depressed states

Pregnant women filled out a self-rating depression scale (SDS), the Self-Rating Anxiety Scale (SAS), and Pittsburgh Sleep Quality Index (PSQI) during the second trimester and third trimester, respectively. The SDS consists of 20 items included two items of psycho-emotional symptoms, eight items of physical disorders, two items of psychomotor disorders, and eight items of depressive psychological disorders. The score for each item ranges from 1 to 4. The total score is multiplied by 1.25 to obtain a standard SDS scale. Standard score ≥ 53 indicates depression. The total reliability coefficient of SDS is 0.784 (Cronbach’s alpha) in Chinese women of rural area, and it has been proven to be a valid and efficient tool for screening depression in Chinese population [24].

The SAS is a 20-item self-administrated scale to measure anxiety. Each question is scored on a scale of 1 to 4 (rarely, sometimes, frequently, and always). The total score ranges between 20 and 80, which is multiplied by 1.25 to obtain a standard SAS scale. Standard score ≥ 50 indicates anxiety. The SAS is a valid tool with good internal consistency (Cronbach’s alpha, 0.897) and widely used to screen anxiety [25].

Sleep quality during pregnancy was assessed using PSQI. It consists of 19 self-rated questions and 5 questions rated by the bed partner. The 19 items are grouped into scores with the seven following components: subjective sleep quality, sleep latency, sleep duration, sleep efficiency, sleep disturbances, use of sleep medication, and daytime dysfunction. These component scores are added to a global PSQI score with a range of 0 to 21, with higher scores indicating worse sleep quality. PSQI score above 5 is abnormal [26].

### Diagnosis of SGA birth

The diagnosis of SGA is a birth weight of infants less than the 10th percentile for gestational age, using the Chinese neonatal birth weight curve [27].

### Placenta sample collection

Approximately 1 g tissue from the maternal side of the placenta in each participant was obtained immediately after delivery. Samples of placental parenchyma were carefully dissected by trained research assistants to assure the maternal decidua was separated from the sample, from which the DNA extracted was of fetal origin. The samples were snap-frozen in liquid nitrogen, and after at least 1 h later, stored in sample tubes at 80℃ for further examination.

### Assessment of methylation levels of the promoter regions of placental CRH, HSD11β2, SLC16A10, DIO3, and MTNR1B genes by NGS-BSP

Methylation changes in the promoter region of CRH, HSD11β2, SLC16A10, DIO3, and MTNR1B genes were assessed by next generation sequencing-based bisulfite sequencing PCR (NGS-BSP). BSP primers were designed using the online MethPrimer website. DNA samples were extracted using the QI Amp DNA Mini Kit (Qiagen, Inc.). Purified DNA was quantified using an ND-1000 spectrophotometer (Nanodrop), and DNA samples (1 μg) were bisulfite-modified using an EZ DNA Methylation Kit (Zymo Research). For each sample, BSP products of target genes were generated, pooled equally, and subjected to adaptor ligation. Barcoded libraries from all samples were sequenced on the Illumina HiSeq platform using a paired-end 150 bp strategy. The mean methylation value was used after discarding any outlying values (deviation of ± 10% methylation from the median).

Five amplification assays were designed using EpiDesigner software (www.epidesigner.com). For CRH, the forward primer 5′- GGGGAGTTTTTTTATATAGTTAGTAAGGAGTAA -3′ and reverse 5′-CCACCAACTTTACRTTACCTAAACTACC -3′ amplified a 294 bp region (hg19: chr8: 67,090,208–67,090,502) providing DNA methylation for 12 CpG units. For HSD11β2, the forward primer 5′- GTTAGTTTTTGTTTTAGGTAGGTTTTGTGGT -3′ and reverse 5′- CACAACCGACATCCCGATACCCTTTACTAATC -3′ amplified a 243 bp region (hg19: chr16: 67,464,413–67,464,656) providing DNA methylation for 28 CpG units. For SLC16A10, the forward primer 5′- TTTGTGTTTTTTTTTGAGTAGGAGGAGGTT -3′ and reverse 5′- AAAATCRACACTAAAACCTCTAAATCACAACCCC -3′ amplified a 262 bp region (hg19: chr6: 111,408,489–111,408,751) providing DNA methylation for 23 CpG units. For DIO3, the forward primer 5′- AGGGGGTTTYGGGGTTTGTAGTTATTATGTT -3′ and reverse 5′- CTTAAAAAAATCCAACTTCTAACCATACCACAC -3′ amplified a 328 bp region (hg19: chr14: 102,027,886–102,028,214) providing DNA methylation for 28 CpG units. For MTNR1B, the forward primer 5′- TTGTATAYGYGAGTTGGGTAGGGAAGAGAG -3′ and reverse 5′- CCACCCAAAAAAATCRAAAAATCCTAAAAAACC -3′ amplified a 226 bp region (hg19: chr11: 92,702,783–92,703,009) providing DNA methylation for 27 CpG units.

### Statistical analysis

For continuous variables, the Shapiro–Wilk test was used to determine the normal distribution of the continuous variables, and the Wilcoxon-Mann–Whitney U-test was conducted for skewed distributions (presented as the median and the interquartile range). Descriptive statistics for categorical variables were reported as frequency (percentage) and compared using the Pearson chi-square test or Fisher's exact test, as appropriate. Methylation levels in CpG sites of placental target genes below 0.01 were excluded from the analyses, and the remaining CpG units were used in the analyses. According to the exposure (depressed emotion of mother) and outcome (SGA birth), the placenta samples were divided into 4 groups: (1) depression mother with SGA (D-S), (2) depression mother without SGA (D-NS), (3) non-depression mother with SGA (ND-S), and (4) non-depression mother without SGA (ND-NS). ANOVA tests and t-tests were used to analyze the differences of methylation of CRH, HSD11β2, SLC16A10, DIO3, and MTNR1B in placenta among the four groups. Logistic regression was used to examine the risk factors of SGA birth, the correlation between any two independent variables in the logistic regression equation should be less than 0.8. Skewed distribution data were log-transformed to obtain a normal distribution. Odds ratios with 95% CIs were calculated. All statistical analyses were performed with SPSS version 24.0, and a *p*-value < 0.05 was considered statistically significant.

## Results

### Occurrence and characteristics of SGA birth in this study population

A total of 345 pregnant women were enrolled in this cohort study. There were 97 (28.1%) and 95 (27.5%) pregnant women who had depression in the second trimester and third trimester, respectively. Thirty-five pregnant women had an SGA birth. The incidence of SGA births in this prospective cohort was 10.1%. In order to analyze the effect of clinical variables on SGA births, we examined the association between characteristics and clinical variables in the pregnant women with and without SGA births (see in Table 1).

**Table 1:** Characteristics of pregnant women with and without SGA birth

	**All**	**With SGA**	**Without SGA**	**P**
	345	35 (10.1%)	310 (89.9%)	
**Maternal age**	29 (27–33)	29 (27–33)	28 (26–32)	0.22
**BMI**	20.1 (18.5–22.1)	19.2 (17.3–21.0)	20.3 (18.6–22.1)	0.07
**Passive smoking**				0.43
Yes	218	20 (9.2%)	198 (90.8)	
No	127	15 (11.8%)	112 (88.2%)	
**Adverse pregnancy history**				0.99
Yes	24	2 (8.3%)	22 (91.7%)	
No	321	33 (10.3%)	288 (89.7%)	
**Education level**				0.15
Non-high education	225	19 (8.4%)	206 (91.6%)	
High education	120	16 (13.3%)	104 (86.7%)	
**Economic level**				0.01
Low income	131	20 (15.3%)	111 (84.7%)	
Non-low income	214	15 (7.0%)	199 (93.0%)	
**Vegetarianism**				0.99
Yes	41	4 (9.8%)	37 (90.2%)	
No	304	31 (10.2%)	273 (89.8%)	
**Pregnancy anemia**				0.25
Yes	89	6 (6.7%)	83 (93.3%)	
No	256	29 (11.3%)	227 (88.7%)	
**Pregnancy hypothyroidism**				0.08
Yes	4	1 (25%)	3 (75%)	
No	341	34 (10%)	307 (90%)	
**Pregnancy diabetes**				0.75
Yes	66	6 (9.1%)	60 (90.9%)	
No	279	29 (10.4%)	250 (89.6%)	
**HDCP**				0.02
Yes	19	5 (26.3%)	14 (73.7%)	
No	326	30 (9.2%)	296 (90.8%)	
**Second trimester** **Maternal depression**				0.04
Yes	97	17 (17.5%)	80 (82.5%)	
No	248	18 (7.3%)	126 (92.4%)	
**Third trimester** **Maternal depression**				0.39
Yes	95	12 (12.6%)	83 (87.4%)	
No	250	23 (9.2%)	227 (90.8%)	
**Delivery method**				0.72
Vaginal	197	21 (10.7%)	176 (89.3%)	
Cesarean	148	14 (9.5%)	134 (90.5%)	
**Preterm delivery**				0.09
Yes	25	5 (20.0%)	20 (80.0%)	
No	320	30 (9.4%)	290 (90.6%)	
**Uterine infection**				0.78
Yes	12	2 (16.7%)	10 (83.3%)	
No	333	33 (9.9%)	300 (90.1%)	
**Placenta previa**				0.78
Yes	12	2 (16.7%)	10 (83.3%)	
No	333	33 (9.9%)	300 (90.1%)	
**Gestational age**	39.14	38.86	39.29	< 0.001
	(38.43–39.86)	(37.43–39.29)	(38.57–40.00)	
**Birth weight**	3.15 (2.95–3.40)	2.65 (2.40–2.75)	3.20 (3.00–3.45)	0.006
**Gender of infants**				0.47
Male	177	20 (11.3%)	157 (88.7%)	
Female	168	15 (8.9%)	153 (91.1%)	

*HDCP* hypertensive disorder complicating pregnancy, *SGA* small for gestational age

### Risk factors for preterm and SGA births

According to logistic regression analysis, the risk factors for SGA birth were low BMI of pregnancy women (RR = 0.71, 95%CI = 0.54 ~ 0.92), HDCP (RR = 4.7, 95%CI = 1.18 ~ 18.72), and second trimester maternal depression (RR = 3.71, 95%CI = 1.31 ~ 12.16) (see in Table 2). The incidence of SGA births was 9.2% in pregnant women who did not have HDCP, but it was increased to 26.3% in those who had HDCP. Pregnant women who did not have depression had 7.3% SGA births, but the incidence of SGA births rose to 17.5% in pregnant women who had depression in the second trimester (see in Table 1).

**Table 2:** Logistic regression of the risk factors for SGA birth

Variables	RR	95%CI	*P*-value
BMI of mothers	0.71	0.54–0.92	0.010
Economic level	0.54	0.19–1.51	0.239
Pregnancy hypothyroidism	2.15	0.48–8.36	0.642
HDCP	4.70	1.18–18.72	0.028
Preterm delivery	5.74	0.74–44.68	0.095
Maternal depression in the second trimester	3.71	1.13–12.16	0.031

*BMI* body-mass index, *HDCP* hypertensive disorder complicating pregnancy, *RR* relative risk, *SGA* small for gestational age

### Methylation levels of the promoter regions of placental CRH and DIO3 genes in mothers with and without depression in the second trimester

Due to the expense of NGS-BSP, we used a nested case–control study to select specimens. We found depression of mother in the second trimester was one of the risk factors of SGA, and then the placentas were divided into four groups (1) depression in the second trimester with SGA (D-S), (2) depression in the second trimester without SGA (D-NS), (3) non-depression in the second trimester with SGA (ND-S), and (4) non-depression in the second trimester without SGA (ND-NS). Each groups have 17 samples, because there was 17 pregnancy women who had depressed emotion in the second trimester with SGA birth. We compared some factors that may affect the SGA birth, including fetal sex, gestational age, HDCP, BMI of mother, age of mother. There was no difference of the above-mentioned factors among the above four groups (see in Table 3).

**Table 3:** Characteristics of samples for NGS-BSP

	SGA (*N* = 34)	Without SGA (*N* = 34)	*P* value
Age of mother	28(26–33.5)	30(28–31)	0.61
BMI of mother	20.1 ± 1.5	20.1 ± 2.6	0.99
Gestational age	38.7(37.1–39.5)	38.3(37.2–39.7)	0.81
Fetal sex			1
Female	15	15	
Male	19	19	
HDCP			1
Yes	5	5	
No	29	29	

*BMI* body-mass index, *HDCP* hypertensive disorder complicating pregnancy, *SGA* small for gestational age

**Fig. 1:**
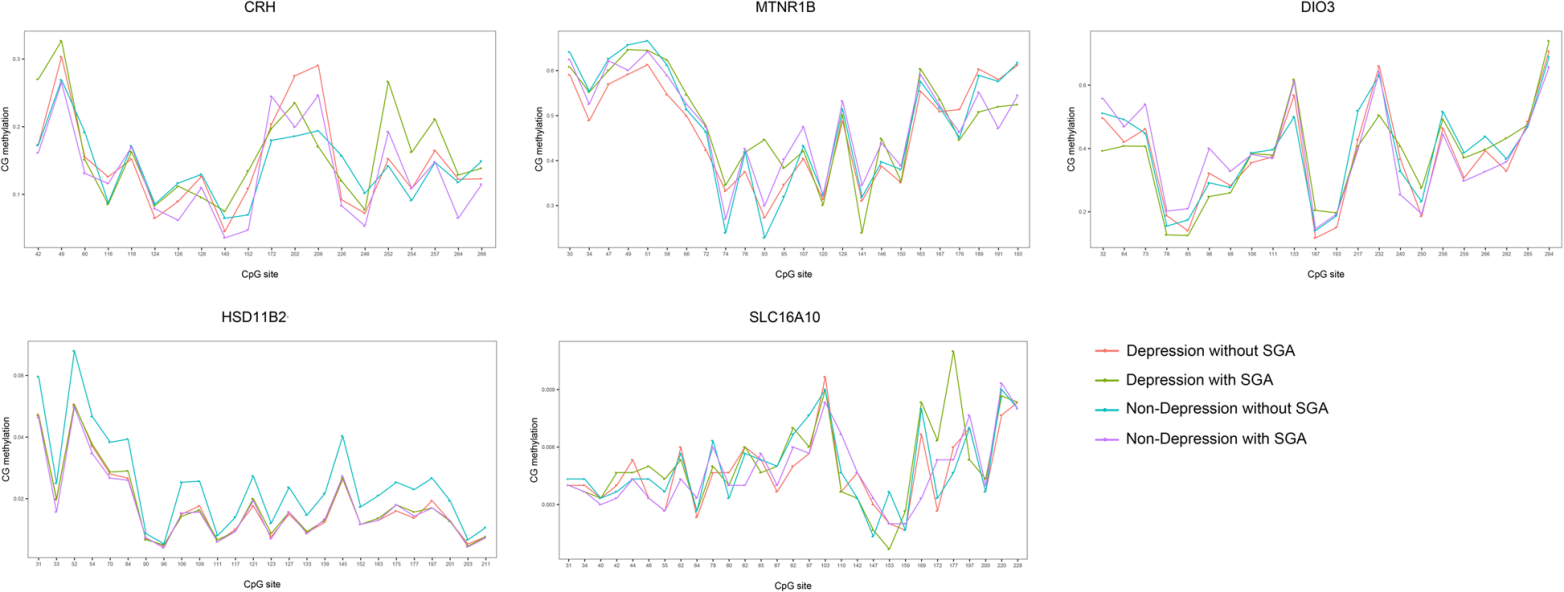
Methylation of CRH, HSD11β2, SLC16A10, DIO3, and MTNR1B genes in the placenta. Methylation measured across difference CpG sites in the promoter regions of CRH, HSD11β2, SLC16A10, DIO3, and MTNR1B genes in the placenta are presented. The samples were divided into four groups, including mother had depression in the second trimester without SGA, mother had depression in the second trimester with SGA, mother did not had depression in the second trimester without SGA, mother did not had depression in the second trimester with SGA

**Fig. 2:**
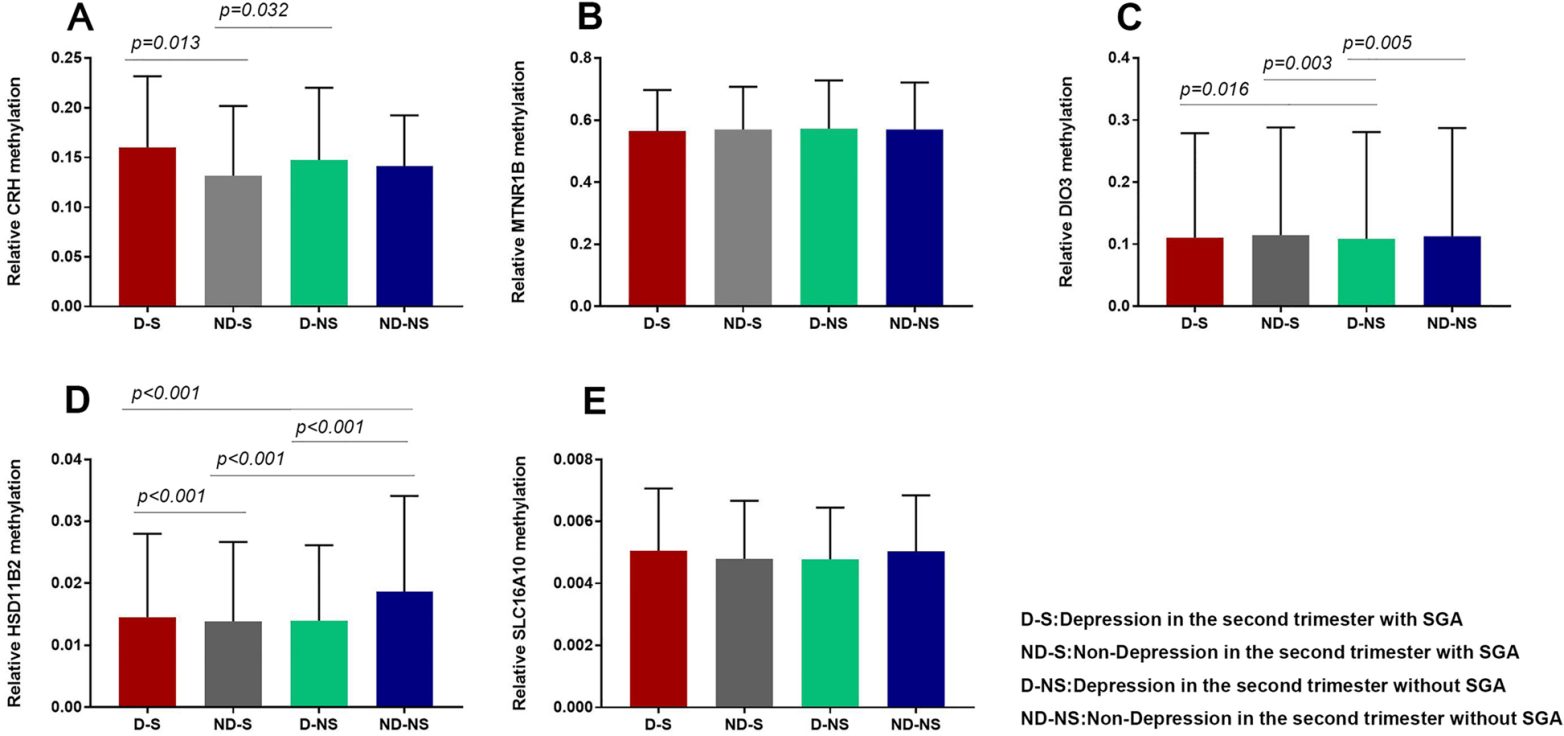
Comparison of placental gene (CRH, HSD11β2, DIO3, SLC16A10, and MTNR1B) methylation levels in four groups. The samples were divided into four groups, including depression in the second trimester with SGA (D-S), depression in the second trimester without SGA (D-NS), non-depression in the second trimester with SGA (ND-S), and non-depression in the second trimester without SGA (ND-NS). Methylation levels of CRH (**A**), MTNR1B (**B**), DIO3 (**C**), HSD11β2 (**D**), SLC16A10 (**E**) gene in these four groups were compared with each other

**Fig. 3:**
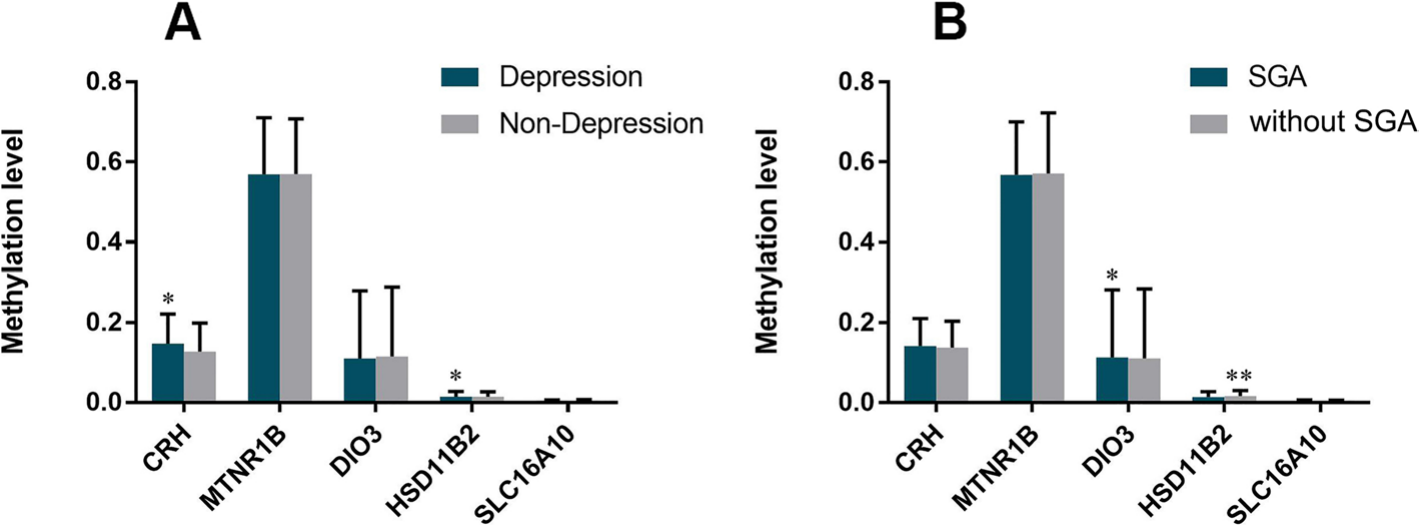
Comparison methylation level of CRH, HSD11β2, DIO3, SLC16A10, MTNR1B according to depression of mother and SGA birth. Comparison methylation level of CRH, HSD11β2, DIO3, SLC16A10, MTNR1B between mother with and without depression in the second trimester (**A**), as well as mother with and without SGA birth (**B**). The paired t-test was used for statistical analysis (*: *p* < 0.05; **: *p* < 0.01)

Methylation measured across difference CpG sites in the promoter regions of CRH, HSD11β2, SLC16A10, DIO3, and MTNR1B genes in the placenta are presented in Fig. 1. We found that the CRH methylation levels were higher in D-S group and D-NS group than ND-S group (Fig. 2A). DIO3 methylation levels were higher in D-S group, ND-S group, and ND-NS group than D-NS group (Fig. 2C). Furthermore, HSD11β2 methylation levels were higher in ND-NS than other groups, and same phenomenon could be observed between D-NS group and ND-S group (Fig. 2D). The methylation levels of MTNR1B and SLC16A10 did not show difference between these four groups (Fig. 2B and E). We found that the CRH and HSD11β2 methylation levels were higher in depression group than non-depression group (see Fig. 3A). DIO3 methylation levels were higher in SGA group than non-SGA group (Fig. 3B). But HSD11β2 methylation levels were lower in SGA group than non-SGA group (Fig. 3B). Higher methylation levels of CRH correlated with higher methylation levels of DIO3 (*r* = 0.658, *p* = 0.02) in placenta. The above results suggested that maternal depression in the second trimester may lead to the changes of methylation in the promoter region of CRH and HSD11β2 gene, while the changes of methylation of DIO3 in subsequent could related to the SGA birth.

## Discussion

In our study, the occurrence of depression during pregnancy is in line with other findings that reported the prevalence of prenatal depression to range from 10 to 40% [28]. And the incidence of SGA in our cohort basically consistent with the SGA occurrence in Chinese study [29]. Moreover, we found low maternal BMI and HDCP are the major risk factors for SGA [30]. The above results indicate that the study population is representative. The placenta is responsible for maintaining and regulating the pregnancy stage as an endocrine organ present at the maternal–fetal interface [15, 16]. It is essential for fetal growth during the entire intrauterine development, and it considered be important and readily available tissue to assess intrauterine exposure and pathology datum [15, 16]. Study placental epigenetic changes which directly affect fetal growth can help elucidate the relationship between SGA and maternal depression during pregnancy.

Our results showed that the methylation level of CRH gene was higher in the D-S group and D-NS group than in the ND-S group, and similar results were observed in comparison of depression group and non-depression group. Consistent with the study about mothers suffer from perinatal stress and war trauma, stress exposures were associated with increased methylation at all CRH sites in cord blood [31]. When suffer from stress, cortisol levels in the blood elevates [4]. However, the increase of CRH methylation level in blood leads to the decrease of serum CRH level, thereby reducing the secretion of cortisol through HPA axis, so that the body avoids exposure to excessive cortisol. In our opinion, it may be the body's self-protective mechanism. In this study, we detected the methylation of CRH in the placenta, which the function of CRH here is difference from that in the blood. Placenta CRH is the major mediator of adaptive response to stressors during pregnancy [5]. CRH can induce the expression of HSD11β2 in isolated trophoblasts [5]. We found the methylation pf CRH and HSD11β2 were both increased in the placenta of pregnancy women who suffer from depressed emotion in the second trimester, which can explain the relationship between CRH and HSD11β2 in the placenta.

The placenta regulates fetal exposure to glucocorticoids by converting active glucocorticoids into their inactive metabolites via HSD11β2 [6]. With the increased methylation of HSD11β2 in the placenta of pregnancy women who had depressed emotion in the second trimester, the expression of HSD11β2 was decreased. And then it could result in an influx of glucocorticoids into the fetal circulation [32]. Overexposure of the fetus to glucocorticoids during pregnancy reduces birth weight [32]. However, how excessive glucocorticoids in the fetus affect birth weight of fetus is unknown.

The hormones produced by the HPA axis and HPT axis counter-regulate one another [33]. Thus, dysregulation of either axis is likely to result in an imbalance of the other. It is for this reason that maternal stress and stress-related biological processes during pregnancy may be important modulators of thyroid function in fetus [33]. Different tissues modulate the impact of circulating THs according to their current needs via three iodothyronine deiodinases (D1, D2, and D3). D3, which is expressed in the organs of the fetus-maternal interface, catalyzes only inner ring deiodination and mainly deactivates T3 and T4 to inactive iodothyronines, such as rT3 [34]. Glucocorticoids generally inhibit thyroid function [33]. Maternal or fetal administration of glucocorticoids in late pregnancy increases plasma T3 and rT3 but not T4 concentrations, which is probably due to a decrease in placental D3 activity [35]. We postulate that increases in the promoter region of the placental DIO3 may result from the increase of cortisol levels due to maternal depression in the second trimester. And our study results also showed that the genetic programming of the HPT axis is an important regulation way for fetus growth in intrauterine, which caused by the abnormal HPA axis due to the depressed emotion of mother in the second trimester of pregnancy. However, the methylation level of HSD11β2 in the placenta of mother with SGA birth is opposite to that in the placenta of mother with depression in the second trimester. We believe that this was another evidence of reciprocal regulation of the fetal HPT and HPA axes. When maternal depression during pregnancy interfered with fetal growth, placental DIO3 methylation increased and DIO3 activity decreased, the fetal HPT axis was influenced. The fetal HPT axis can also regulate to the HPA axis, increasing HSD11β2 expression and adjusting cortisol concentrations to avoid exposure to excessive cortisol caused by maternal depression, which may be a mechanism of body self-regulation in fetus. Therefore, the methylation of placenta HSD11β2 have been explored for a potential programming effect of the fetal HPA axis [36].

Both brain and placenta MTNR1B gene encode high-affinity receptors of melatonin, which expresses in the placenta during pregnancy and promotes trophoblast syncytium formation [37]. However, the difference of MTNR1B methylation did not observe in our study, probably because it already matched sleep quality in different groups before gene detection. SLC16A10 gene encode monocarboxylate transporters that highly active and specific for thyroid hormones [10]. Low methylation of SLC16A10 gene was observed in this study and showed no difference between groups. It could be explained in another human study, which showed no statistically significant differences with the SGA samples compared to normal birth weight samples at both the mRNA and protein levels in SLC16A10 gene, since it is widely expressed in villous and extra villous trophoblasts from an early gestational age [38].

## Conclusions

In summary, the results in this study suggest that maternal depression in the second trimester could disrupt the HPA axis, but also influence the regulation of HPT axis and then result in a high incidence of SGA. The mechanism may be related to the changes of methylation in the promoter region of the placental DIO3, HSD11β2 and CRH genes. Because epigenetic alterations can result in long-term consequences of behavior and endocrine response in the offsprings, screening those children, early recognition of risk factors and intervention should be a consideration. The healthcare professionals can use the evidence of this study to improve the maternal and neonatal care. On the one hand, obstetricians should pay attention to the emotional problems of pregnant women during pregnancy, which may affect fetal development even if depression is not diagnosed. On the other hand, pediatricians should pay attention to babies whose mothers had depressed emotion during pregnancy, because the effects of maternal depressed emotion are not only result in SGA birth but may have long-term effects on the growth and development of offspring due to the epigenetic changes. However, small sample size may lead to type II errors, and we should increase the sample size for a more valid argument in the future research.

## Data Availability

The data in this study are available from the corresponding author on reasonable request.

## Abbreviations

BMI: Body mass index
CRH: Corticotropin-releasing hormone
DIO3: Deiodinase 3
HDCP: Hypertensive disorder complicating pregnancy
HPA: Hypothalamic–pituitary–adrenal
HPT: Hypothalamus-pituitary-thyroid
HSD11β2: 11β-Hydroxysteroid dehydrogenase type 2
MTNR1B: Melatonin receptor 1B
NGS-BSP: Generation sequencing based on bisulfite sequencing PCR
SDS: Self-rating depression scale
SGA: Small for gestational age
SLC16A10: Solute carrier family 16 member 10
